# Artificial Intelligence in Echocardiography: The Time is Now

**DOI:** 10.31083/j.rcm2308256

**Published:** 2022-07-19

**Authors:** Amro Sehly, Biyanka Jaltotage, Albert He, Andrew Maiorana, Abdul Rahman Ihdayhid, Adil Rajwani, Girish Dwivedi

**Affiliations:** ^1^Department of Cardiology, Fiona Stanley Hospital, WA 6150 Murdoch, Australia; ^2^Department of Allied Health, Fiona Stanley Hospital, WA 6150 Murdoch, Australia; ^3^Curtin School of Allied Health, Curtin University, WA 6102 Bentley, Australia; ^4^Harry Perkins Institute of Medical Research, WA 6150 Murdoch, Australia; ^5^School of Medicine, The University of Western Australia, WA 6009 Crawley, Australia; ^6^School of Medicine, Curtin University, WA 6102 Bentley, Australia; ^7^Department of Cardiology, Royal Perth Hospital, WA 6000 Perth, Australia

**Keywords:** artificial intelligence, deep learning, echocardiography, machine learning

## Abstract

Artificial Intelligence (AI) has impacted every aspect of clinical medicine, and is predicted to revolutionise diagnosis, treatment and patient care. Through novel machine learning (ML) and deep learning (DL) techniques, AI has made significant grounds in cardiology and cardiac investigations, including echocardiography. Echocardiography is a ubiquitous tool that remains first-line for the evaluation of many cardiovascular diseases, with large data sets, objective parameters, widespread availability and an excellent safety profile, it represents the perfect candidate for AI advancement. As such, AI has firmly made its stamp on echocardiography, showing great promise in training, image acquisition, interpretation and analysis, diagnostics, prognostication and phenotype development. However, there remain significant barriers in real-world clinical application and uptake of AI derived algorithms in echocardiography, most importantly being the lack of clinical outcome studies. While AI has been shown to match or even best its human counterparts, an improvement in real world outcomes remains to be established. There are also legal and ethical concerns that hinder its progress. Large outcome focused trials and a collaborative multi-disciplinary effort will be necessary to push AI into the clinical workspace. Despite this, current and emerging trials suggest that these systems will undoubtedly transform echocardiography, improving clinical utility, efficiency and training.

## 1. Introduction

Echocardiography stands at the pinnacle of cardiac investigations. It provides a rapid, non-invasive and accurate assessment of biventricular structure and function, pulmonary pressures, valvular function and intracardiac shunts [1]. Its widespread availability, low cost and safety profile have pushed it beyond the confines of specialist cardiologists, and is now a crucial instrument for all clinicians [2, 3]. With the ongoing rise in prevalence of cardiovascular disease, the utilisation of echocardiography is expected to increase worldwide, with US trend data already showing an annual growth of 3.41% [4, 5].

Echocardiography isn’t without its pitfalls. This includes subjective interpretation of data points, resulting in poor inter and intra-observer correlation and a reliance on operator dependant acquisition of images and key measurements that dictate results [6]. Additionally, intensive training is required to develop expert skills to perform and interpret echocardiography [7]. Reporting echocardiography studies can also be demanding, limiting workflow in smaller centres with fewer trained staff. Lastly, a range of significant pathologies up to now have been better assessed with other modalities, such as infiltrative cardiomyopathies with cardiovascular magnetic resonance (CMR) imaging, or coronary artery disease with coronary computed tomographic angiography (CCTA) [8]. Artificial intelligence (AI) based technology has emerged to meet these challenges. Echocardiography is uniquely positioned to lend itself to AI, with huge data sets, large volumes of patients and well-established objective parameters for disease pathology. It will inevitably be incorporated into all forms of cardiac imaging with a significant impact on guiding diagnosis and clinical decision making [9, 10].

AI has made its first foray into echocardiography with notable results, from acquisition to interpretation. AI has been shown to be of benefit in educating and training health care staff in image acquisition [11, 12]. Studies involving AI models that assist with echocardiography interpretation have demonstrated a significant reduction in inter-observer variability and improved reproducibility [13]. Whilst other studies have used AI algorithms to improve the diagnostic utility of echocardiography in pathologies that currently require CMR for diagnosis [14].

The purpose of this review is to summarize the recent discoveries and advances of AI in echocardiography, its future prospects and the current pitfalls and limitations of its use.

## 2. Artificial Intelligence

AI is defined as the theory and development of computer systems able to perform tasks normally requiring human intelligence. Machine learning (ML) is a subfield of AI which allows for the analysis of vast quantities of data through computing and statistical algorithms [15]. This system infers relationships between data to assess which data points have the highest predictive power [15]. Through these techniques, ML models are able to provide predictions based on unseen data [15].

**Fig. 1.**
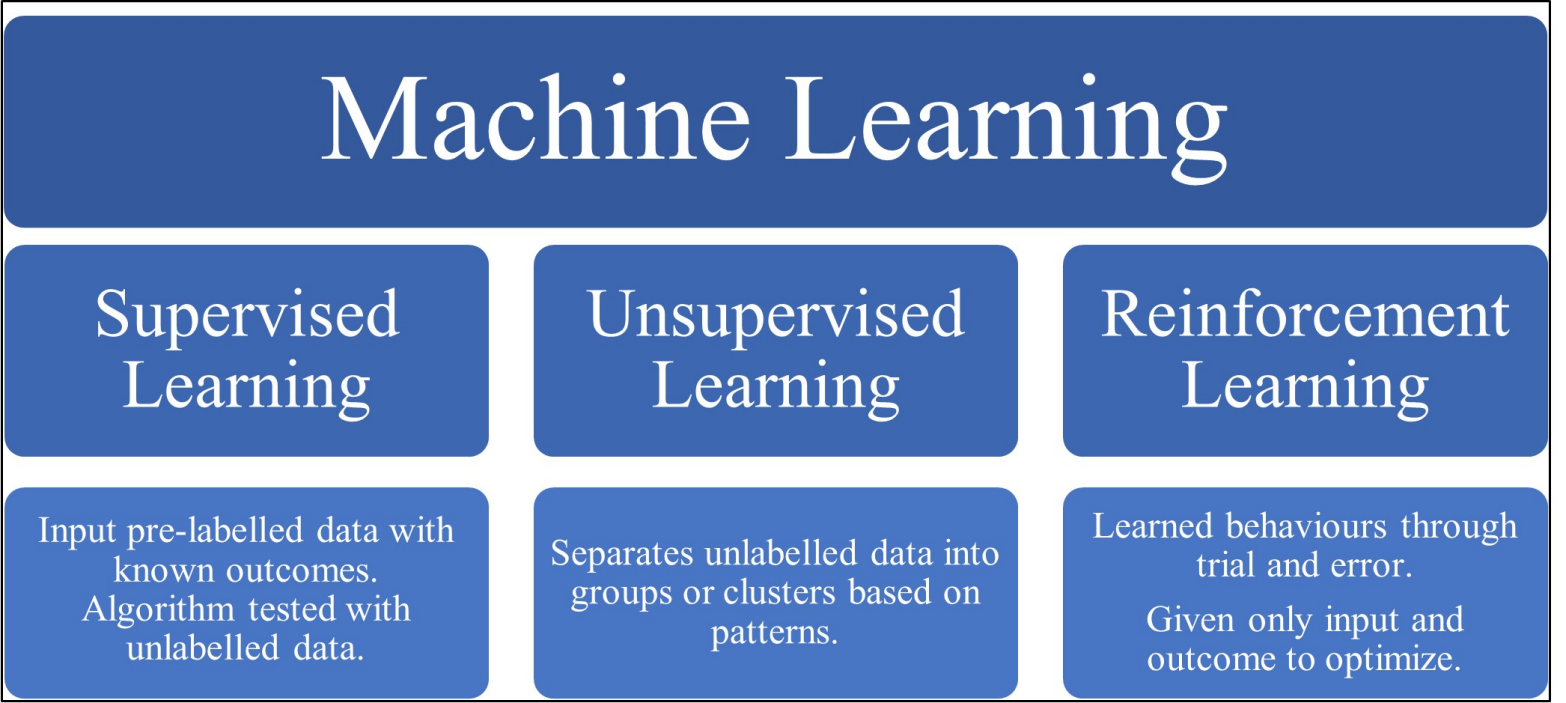
**A diagram depicting the common types of machine learning along with a brief explanation of each type**.

ML can be classified into three categories, supervised, unsupervised and reinforcement learning (see Fig. 1) [16]. Firstly, in supervised learning the machine is ‘taught’ to classify data by providing it with a dataset of labelled data [17]. This is then tested with a new unlabelled dataset, allowing an assessment of the accuracy of the model [17]. Unsupervised learning focuses on discovering new patterns and associations between variables using unlabelled datasets [10]. Allowing data exploration and the generation of novel hypotheses, including the development of refined and individualised disease phenotypes [10, 15]. Finally, reinforcement learning algorithms are learned behaviours through trial and error, given only input data and an outcome to optimize [15]. A popular culture breakthrough example of this was when a learning model was used to beat the high score on 49 Atari video games provided with only video input and the game’s final score [18].

Deep learning (DL) is a subset of ML consisting of networks of nodes that mimic the brain, called artificial neural networks [10, 15]. These nodes interconnect; the first level of “input” nodes point into another layer of nodes in the network called “hidden layers”, these then connect to an “output layer” of nodes [10, 15]. Two of the most common forms of DL include convolutional neural networks (CNN) and recurrent neural networks [15]. CNN can be used to process two-dimensional (2D) image based data into multiple layers, proving invaluable in echocardiography, radiology, pathology and dermatology [10, 17, 19, 20]. Conversely, recurrent neural networks are well suited for sequential data such as speech and language and thus is used in machine interpretation of text and speech recognition [15, 21]. DL methods have also been used in drug development, with work by Jamshidi *et al*. [22] demonstrating a conceptual framework for COVID-19 drug discovery.

## 3. Artificial Intelligence in Echocardiography 

### 3.1 Image Acquisition and Recognition of Views

A transthoracic echocardiography study requires accurate image acquisition of the standard windows, parasternal long and short axis views, apical views and subcostal views [1]. These views and key measurements are captured, with varying echocardiographic assessments applied, such as M-mode, colour Doppler, tissue Doppler, pulse-wave Doppler and continuous-wave Doppler [1]. Combining these assessments allows for precise evaluation of the three-dimensional (3D) structures of the heart to be extracted from 2D cross-sectional images [10]. All of this is predicated on accurate image acquisition, a limiting factor of echocardiography. A number of factors affect image quality such as the extensive training required to produce a high quality transthoracic echocardiographic study, and common conditions such as obesity and chronic airway disease that frequently limit echocardiographic views [7]. Additionally, while the general approach to an echocardiographic study is standardised, no two patients are identical in terms of image acquisition, the natural variability of human anatomy demands adaptive approaches for high quality images, necessitating experienced hands [7].

AI has already entered the clinical workspace with commercially available software for echocardiographic image acquisition and interpretation [23, 24]. These AI derived models provide user guidance on image acquisition, with clear instructions on probe positioning and alerting the user of poor image quality, thereby providing a degree of training and self-improvement [23, 24].

Narang *et al*. [11] recently demonstrated the use of a DL algorithm in guiding eight nurses with no prior ultrasonography experience in the acquisition of echocardiograms for limited diagnostic use. In this study, expert echocardiographers blindly reviewed the scans and felt they were of diagnostic quality for key parameters including left ventricular (LV) size and function, right ventricular (RV) size and function and the presence of a pericardial effusion [11]. Another study by Schneider *et al*. [12] used a ML algorithm to train 19 echo-naïve first-year medical students to acquire diagnostic echocardiography images, the AI algorithm then obtained LV ejection fraction (LVEF) values from these images. These were compared with images obtained by three experts, the novices were able to attain at least one of three views 91% of the time and an excellent agreement between the novice LVEF and the expert derived LVEF was found (correlation coefficient of 0.92) [12].

AI has also made advancements in accurately recognising and fitting echocardiographic views, Madani *et al*. [25] used CNN to develop a model to classify 15 standard views based on 267 labelled studies with real-world clinical variation. Their model was able to classify the views with a 97.8% overall test accuracy for video views [25]. Furthermore, on still images an overall accuracy of 91.7% was achieved, significantly (*p* = 0.003) better than the result for board-certified echocardiographers at 79.4% [25]. More importantly, recognition of these views occurred very fast at an average of 21 milliseconds per view, highlighting the unmatched efficiency that AI offers [25].

Zhang *et al*. [26] trained and evaluated CNN network models for multiple tasks in echocardiography including image classification of 23 standard views and segmentation. This ten year study used 14,035 echocardiograms to train the models that had the capacity for interpretation and diagnosis of pathology, a truly comprehensive application of AI in echocardiography [26]. With regards to image identification, the models were able to achieve accuracies of 84% at an individual image level, with accuracies of up to 96% in certain views [26]. Although less accurate than other models, this group was able to incorporate it effectively into an echocardiographic workflow from image acquisition and interpretation to diagnosis [26].

### 3.2 Image Analysis and Interpretation

#### 3.2.1 LV Systolic Function

LV function comprises one of the most important components of an echocardiogram study and carries significant prognostic value [1, 10]. LV systolic and diastolic function underpin the diagnosis of heart failure, current assessments are subject to significant inter-observer variability and poor reproducibility [6, 10]. LVEF is the most used metric in assessing LV function [1]. A multitude of techniques exist for LVEF assessment, the modified Simpson’s biplane is one such frequently used method, requiring manual tracing of end-systolic and end-diastolic perimeters of the LV in the apical four and two chamber views to calculate LVEF [1, 27]. This technique can be challenging due to the reliance on good quality apical views and the time taken to trace the LV which itself is an error prone process [28]. Despite the modified Simpson’s biplane being a more robust assessment of LVEF when compared to other techniques, it is still based on the assumption that the LV is comprised of cylindrical disks and does not take into account variability in structure and shape which can lead to poor correlation with gold standard CMR [1, 27, 28].

Leclerc *et al*. [29] used an encoder-decoder-based CNN DL model to segment and analyse 500 echocardiogram studies with apical four and two chamber views, measuring end-diastolic, end-systolic LV volumes and LVEF. Their model was able to outperform non-DL methods and accurately reproduced expert analysis data, with a mean correlation of 0.95 [29]. The reproducibility of this model was also superior to inter-observer scores for conventional methods [29].

Another group designed a video-based DL algorithm, EchoNet-Dynamic, to rapidly and accurately assess LVEF using only apical four chamber views and in one cardiac cycle [30]. Ouyang *et al*. [30] trained the CNN model with 10030 apical four chamber echocardiogram videos and was able to predict ejection fraction (EF) with a mean absolute error of 4.1%, reliably classifying heart failure with reduced EF with an area under the curve of 0.97. This was done in real time, taking approximately 1.6 seconds per cardiac cycle, much more rapid than human assessment [30]. Moreover, the model was shown to have minimal variation of assessing LVEF on repeat testing as compared with two trained sonographers in a cohort of 55 patients, with a median difference of 2.6% vs. 5.2%, *p *< 0.001 [30]. Notably, they did not exclude studies with poor image quality [30].

Jafari *et al*. [31] took this one step further, by designing a real-team mobile point-of-care ultrasound software to assess LVEF. This DL based system operates on android based mobile devices and simultaneously recognises, segments and analyses LV function in real time using both apical four and two chamber views [31]. This was tested on 427 patients, resulting in accurate results for LVEF, with a median absolute error of 6.2% compared to expert cardiologist annotations and measurements [31]. This shows the untapped potential of AI in accurate and rapid assessment of LVEF in mobile point-of care ultrasounds.

#### 3.2.2 LV Strain

Systolic strain is the deformation that occurs as a consequence of myocardial contraction [32]. It provides novel information on the movement of the myocardium that escapes conventional echocardiography, including the “twisting” and “wringing” motion of the myocardium [32]. This offers more objective measures of LV myocardial dynamics, can help to identify pathological disease patterns and allows for early detection of subclinical ventricular dysfunction. Subsequently, LV strain now has a pivotal role in the diagnosis of cardiac amyloidosis and the early detection of cardiotoxicity secondary to chemotherapeutic agents [32].

Salte *et al*. [33] created a DL model to assess global longitudinal strain using conventional 2D echocardiographic views. This was applied to 200 studies and was compared to the conventional time intensive speckle-tracking software [33]. The AI method successfully performed automatic segmentation and measurements of global longitudinal strain across a variety of cardiac pathologies, showing minimal difference between the methods, with a mean absolute difference of 1.8% [33]. There was also minimal variability in the DL method, and the assessment was rapid, occurring in less than 15 seconds per study with AI compared to 5–10 mins with the conventional method [33]. This work highlights the value of AI for more advanced echocardiographic parameters.

#### 3.2.3 Diastology

The diastolic properties of the heart determine filling during diastole [1, 34]. Diastolic dysfunction is common and thought to affect 5.5% of the general population. Assessment is notoriously difficult, with guidelines recommending the measurement of six different echocardiographic parameters, review of multiple complex flow charts and correlation with a clear clinical picture [27, 34]. Despite these guidelines, the diagnosis can still be challenging and much uncertainty still remains [34]. The risk factors for diastolic dysfunction include obesity, hypertension and diabetes, conditions that are epidemic in our society, and have unsurprisingly precipitated a rise in the prevalence of diastolic dysfunction [35]. AI has provided novel approaches to assessment of diastology (see Table 1, Ref. [36, 37, 38, 39]).

**Table 1.** **Key examples demonstrating the application of AI in the diagnosis of diastolic heart failure**.

Author [Ref]	Study aim	N	Mode of echocardiography	Method of AI	Results
Choi *et al*. [36]	Evaluated diagnostic accuracy of AI assisted clinical decision system for diagnosing diastolic heart failure.	97	2D-echocardiography	Machine learning-driven rule generation and expert driven knowledge acquisition.	Concordance rate for diagnosis of diastolic heart failure compared to heart failure specialists of 99.6%.
Omar *et al*. [37]	Developed and validated an AI model for assessing left ventricular filling pressures.	174	Speckle tracking echocardiography	Machine learning model using random forests, artificial neural networks and support vector machines.	Area under the curve of 0.881 for invasively measured elevated filling pressures.
Pandey *et al*. [38]	Developed and validated a deep learning model that identified distinct patient subgroups with diastolic heart failure.	1242	2D-echocardiography	Unsupervised machine learning and deep learning techniques.	Identified patient with elevated left ventricular filling pressures better than guideline methods, with area under the curve of 0.88 vs. 0.67 (*p* = 0.01).
Chiou *et al*. [39]	Established a rapid pre-screening tool for diastolic heart failure by using AI techniques to detect abnormal patterns in intra-beat dynamic changes.	315	2D-echocardiography	Deep learning and conventional neural network.	Accuracy of 0.91, sensitivity of 0.96 and specificity of 0.85 for detecting diastolic heart failure.

AI, Artificial intelligence.

Recent work has demonstrated the potential value of AI in the evaluation of diastolic function. Choi *et al*. [36] assessed the diagnostic accuracy of a ML model in heart failure, using combined clinical, biochemical and echocardiographic data to derive an algorithm for diagnosis, they found a 99.6% concordance in diagnosing diastolic heart failure as compared to a heart failure specialist. Their algorithm used only LVEF, left atrial volume index and tricuspid regurgitation velocity, a notable improvement from the six parameters normally used in assessing diastology [36]. Using novel speckle tracking echocardiographic derived measurements, Omar *et al*. [37] were able to develop an AI model that accurately predicted increased LV filling pressure, a key parameter of diastolic dysfunction, this was validated with invasively measured raised pulmonary capillary wedge pressure with an area under the curve of 0.88. The AI derived 14 novel variables from speckle tracking echocardiographic measurements of atrioventricular deformation, chamber volume and volume expansion, a further step towards automated diastolic function assessment [37].

**Fig. 2.**
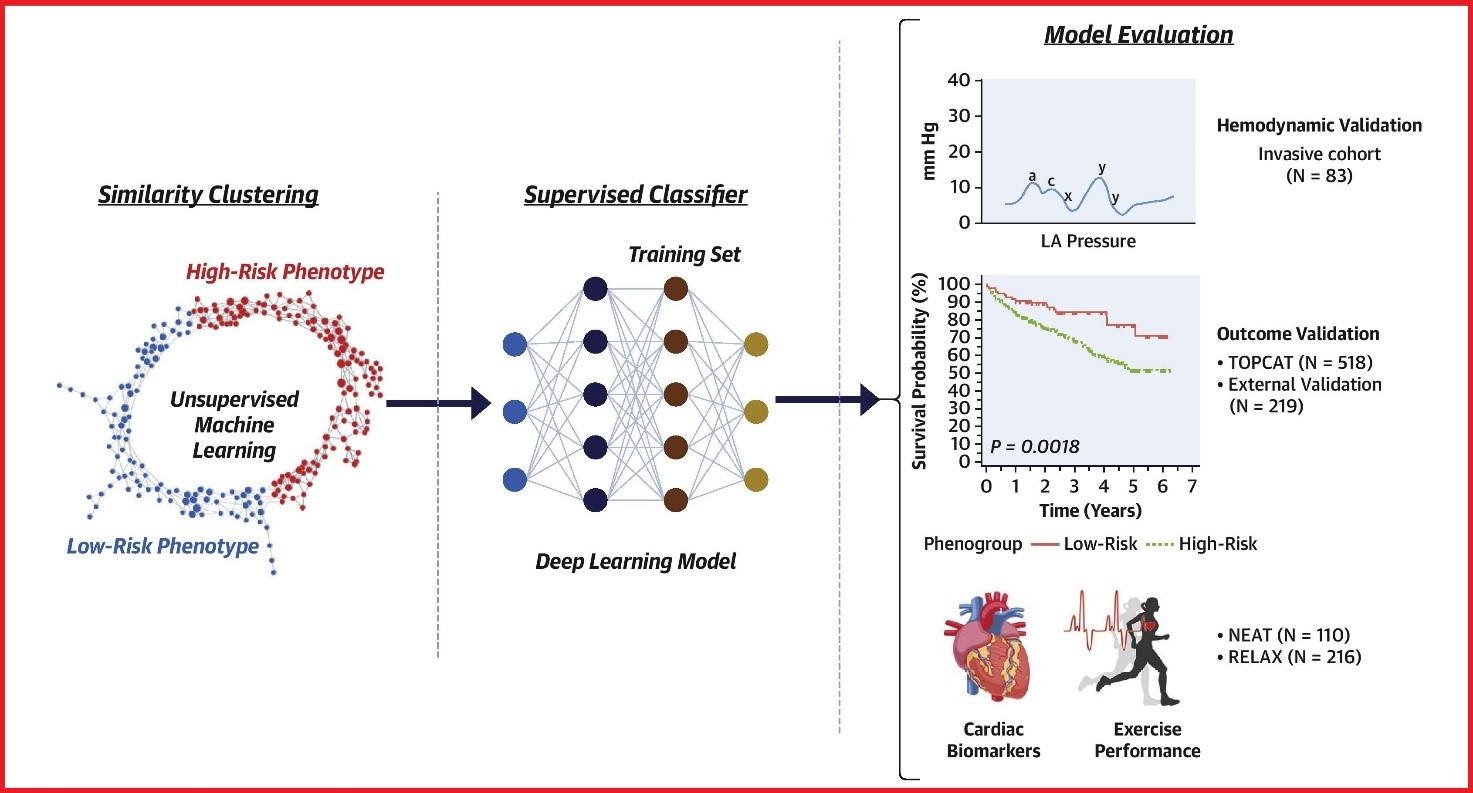
**Pandey *et al*. [38] used unsupervised machine learning and deep learning techniques to develop a new grading of diastolic dysfunction, which outperformed current guideline-based grading in clinical outcomes**. Reproduced with permission from [38].

There have also been endeavours to improve phenotyping of diastolic dysfunction to better predict outcomes; Pandey *et al*. used ML to design a model to more accurately identify patients with elevated LV filling pressure as compared to the American Society of Echocardiography 2016 diastolic guidelines grading system, their work is illustrated in Fig. 2 [38]. Promising work has also been done in developing rapid and accurate screening tools for diastolic dysfunction, Chiou *et al*. [39] developed a pre-screening tool for diastolic heart failure by intra-beat dynamic changes in the LV and left atrium. They used linear signals of LV and left atrium length, area and volume waveforms to determine novel intra-beat dynamic patterns that accurately determine diastolic function, demonstrating an accuracy, sensitivity and specificity of 0.91, 0.96 and 0.85 respectively [39].

#### 3.2.4 RV Function

The RV is challenging to assess but harbours significant prognostic value and clinical relevance [40]. RV function can be affected by congenital heart disease, left-sided heart failure, valvular heart disease, pulmonary hypertension and coronary artery disease [40]. Accurate and reproducible quantification of RV function can be difficult due to its irregular crescent shape, poor echocardiographic visualisation of the RV and inconsistencies in the analysis of RV parameters [41]. AI has shown promise in rapid and accurate assessment of RV function [42, 43].

Zhu *et al*. [42] recently used a novel AI algorithm to assess RV function using 3D echocardiography. The study included 51 participants and compared the results of their AI derived RV function from 3D echocardiography to the gold standard CMR [42]. The AI based 3D echocardiography data showed statistically significant correlation with the corresponding CMR analysis (*p *< 0.05 for all) [42]. The AI based 3D echocardiography RV analysis was completed rapidly at 100 ± 12 seconds in patients with good quality images [42]. The AI algorithm also showed excellent diagnostic performance in identifying RV dysfunction as compared to CMR, with the cut-off RVEF of 43% showing a sensitivity of 94% and specificity of 67% [42].

AI has also been used to develop predictive tools in assessing RV failure post-implantation of a left ventricular assist device (LVAD). A third of all LVAD implantations are complicated by RV failure post-operatively, this is in part due to increased RV preload from the device and excessive leftward shift of the interventricular septum, reducing its contribution to RV contraction [43, 44]. RV failure post LVAD implantation is difficult to predict and is currently based on pre-existing echocardiographic assessment, biomarkers and clinical judgement [43]. Shad *et al*. [43] used video-based DL to predict the likelihood of developing RV failure post device insertion using only 2D echocardiographic data, significantly outperforming a team of human experts at the same task (*p* = 0.016). Although these clinical decisions are never performed with only echocardiographic data, the algorithm clearly demonstrates its worth as an adjunct tool in predicting RV dysfunction, allowing appropriate measures to be taken pre-emptively.

#### 3.2.5 Valvular Function

Echocardiographic assessment of valvular function is a complex field requiring accurate images, precise measurements and a multitude of parameters for assessment [1]. Conventional techniques to assess valvular function are objective, complex and time consuming, making them ideal for AI advancement [1]. For example, work by Moghaddasi *et al*. [45] used ML to develop a novel method for assessing mitral regurgitation, based on image processing techniques and micro-patterns of 2D echocardiographic images. Their technique was able to achieve an excellent sensitivity of 99.38% and specificity of 99.63% for the detection of the different severities of mitral regurgitation [45].

AI has also been used in aortic valve assessment prior to transcatheter aortic valve replacement, Prihadi *et al*. [46] utilised a new AI developed software for accurate measurement of the aortic annulus and root using 3D transoesophageal echocardiography. This is vitally important in accurate sizing and placement of the replacement valve. The results were comparable to assessments with computed tomography, with excellent correlation and low inter and intra-observer variability, paving the way for avoidance of radiation exposure [46]. Queiros *et al*. [47] demonstrated similar findings using a different software, confirming the utility of AI in aortic valve assessment for transcatheter aortic valve replacement [47, 48].

The foray of AI into valvular heart disease remains in its infancy, however significant advancements have already been made, and are predictive of what may still be to come.

#### 3.2.6 Stress Echocardiography

Stress echocardiography is a useful tool to assess for the presence of coronary artery disease, however it suffers from significant inter-observer variability, requires a high level of expertise and has a significant qualitative element in its assessment [6]. Omar *et al*. [49] demonstrated the efficacy of a DL based algorithm that used strain analysis for assessment of stress echocardiograms. This was assessed on a 3D echocardiography dataset of stress echocardiograms, yielding comparable accuracies to standard approaches. This method is limited as it requires the acquisition of strain imaging during stress testing, which can be challenging, despite this it demonstrates the potential of AI in this area [49].

Recently, Upton *et al*. [50] undertook a multi-centre, multi-vendor trial that used a CNN to develop a model that can identify patients with angiographically confirmed prognostic coronary artery disease on stress echocardiograms. The model was then tested on a dataset of 154 stress echocardiograms, showing a specificity of 92.7% and a sensitivity of 84.4% [50]. The authors then put it into practice as an “AI assistant” to be used by clinicians reporting the studies, and it was found to increase the sensitivity for disease detection by 10%, achieving an area under the curve of 0.93 [50]. This demonstrates a practical approach to AI integration into clinical workflow.

#### 3.2.7 Diagnostic Utility

DL methods have been shown to be effective in assessing cardiac diseases and may improve the diagnosis of diseases that are often challenging to diagnose on echocardiography alone (see Table 2, Ref. [26, 51, 52, 53]). Zhang *et al*. [26] used CNNs to develop a fully automated pipeline of echocardiography from image recognition and segmentation to interpretation and disease diagnosis. They were able to detect hypertrophic cardiomyopathy, cardiac amyloidosis and pulmonary arterial hypertension with C statistics of 0.93, 0.87 and 0.85 respectively [26]. Moreover, they were able to successfully integrate this system into the clinical workflow with success, unwrapping the immense potential of AI in echocardiography [26].

**Table 2.** **Key examples demonstrating the application of AI in echocardiographic diagnostics**.

Author [Ref]	Aim of study	N	Mode of echocardiography	Method of AI	Results
Zhang *et al*. [26]	Used AI to build a pipeline for echocardiogram interpretation including disease detection of HCM, cardiac amyloid and PAH.	HCM: 260	2D-echocardiography	Deep learning with convolutional neural networks.	HCM, cardiac amyloid and PAH detected with C-statistics of 0.92, 0.87 and 0.85 respectively.
		Cardiac amyloid: 81	Speckle tracking		
		PAH: 104			
Ghorbani *et al*. [51]	Used AI to identify local cardiac structures, including pacemaker leads.	373	2D-echocardiography	Deep learning with customized convolutional neural network.	Accurately identified pacemaker leads (AUC = 0.89), enlarged left atrium (AUC = 0.86) and left ventricular hypertrophy (AUC = 0.75).
Kusunose *et al*. [52]	Investigated whether a convolutional neural network could provide improved detection of regional wall motion abnormalities.	300	2D-echocardiography	Deep learning with convolutional neural network.	Similar accuracy to cardiologists (AUC 0.99 vs. 0.98, *p* = 0.15). Significantly higher than that of residents (AUC 0.99 vs. 0.90, *p* = 0.002).
Sun *et al*. [53]	Investigated whether TOE assisted with an AI algorithm was superior to TOE alone in diagnosing left atrial appendage thrombi in patients with AF.	130	Transoesophageal echocardiography	Deep learning with artificial neural network.	Improved sensitivity and specificity with AI assisted TOE, higher accuracy rate (0.966 vs. 0.840, *p *< 0.01) and improved AUC (0.932 vs. 0.834).

AI, artificial intelligence; HCM, hypertrophic cardiomyopathy; PAH, pulmonary arterial hypertension; AUC, area under the curve; TOE, transoesophageal echocardiography; AF, atrial fibrillation.

Ghorbani *et al*. [51] developed a customized CNN model to diagnose the presence of pacemaker leads, left atrial enlargement and LV hypertrophy, with an area under the curve of 0.89, 0.86 and 0.75 respectively. The same group had previously used the same model to rapidly and accurately assess LVEF and diagnose heart failure with reduced EF with an area under the curve of 0.97, an assessment done in real time, taking approximately 1.6 seconds per cardiac cycle [30].

Omar *et al*. [49] were able to develop a CNN to automatically assess regional wall motion abnormality by quantifying a cardiac bull’s eye map derived from principal strain analysis during dobutamine stress echocardiograms, effectively diagnosing coronary artery disease. Kusunose *et al*. [52] used a CNN to improve detection of regional wall motion abnormality, demonstrating a similar area under the curve to cardiologists and sonographers (0.99 vs. 0.98 respectively; *p* = 0.15) and a significantly higher area under the curve than junior medical doctors (0.97 vs. 0.83 respectively; *p* = 0.003). ML was also used to develop automated discrimination of hypertrophic cardiomyopathy from physiological hypertrophy seen in athletes using speckle tracking echocardiography, showing increased sensitivity and specificity than traditional markers such as early-to-late diastolic trans-mitral velocity ratio, average early diastolic tissue velocity and strain (*p *< 0.01, *p *< 0.01 and *p* = 0.04 respectively) [13].

Assessment of intracardiac masses by echocardiography is fraught with subjectivity and requires extensive experience, an area ripe for advancement with AI. Strzelecki *et al*. [54] developed and tested an AI derived method for automatic identification of different intracardiac tumour and thrombi with 2D echocardiography, they were able to demonstrate better accuracies, sensitivities and specificities than pre-existing software. Sun *et al*. [53] focused primarily on left atrial and left atrial appendage thrombi, developing a computer-aided diagnostic algorithm to look at transoesophageal echocardiogram images and assessing this in a prospective study. They assessed 130 patients with atrial fibrillation and found their algorithm significantly improved the diagnostic accuracy of transoesophageal echocardiogram for left atrial and left atrial appendage thrombi (*p *< 0.05) [53]. Zhou *et al*. [55] wrote an excellent review highlighting the role of AI in disease diagnosis with echocardiography.

## 4. Future Prospects

### 4.1 Prognostication and Phenotyping

Disease prognostication and personalised risk profiling remains a growing field in cardiology, ML can provide the driving force for the development of novel markers of prognosis in echocardiography. LVEF remains the most commonly used echocardiographic prognostic marker, despite its limitations in this regard [56]. ML has been used to develop more improved and specific prognostic markers, moreover its use has driven the development of new disease phenotypes, delivering more individualised patient care [26, 57, 58, 59].

Samad *et al*. [57] used a non-linear ML model to accurately predict survival using clinical data and echocardiography data, they were able to outperform common clinical risk scores and linear logistical regression model scores (*p *< 0.01). Moreover, they demonstrated that tricuspid regurgitation velocity as a single echocardiographic variable was more predictive of survival than LVEF [57]. Ernande *et al*. [58] utilised unsupervised ML-based cluster analysis of echocardiographic data to derive cardiac phenotypes in patients with type 2 diabetes mellitus, demonstrating different risk profiles and outcomes for each cohort. Omar *et al*. [59] also used unsupervised cluster analysis techniques to assess diastolic dysfunction and derive two phenotypic grades of diastolic heart failure. They showed one cluster as having significantly lower survival free of all-cause mortality, lower cardiac mortality and lower cardiac hospitalizations (*p* = 0.008, 0.026 and 0.09 respectively), while no difference in survival was seen in guideline-based classifications [59]. Zhang *et al*. [26] developed a complete AI based echocardiographic pipeline and used this to assess global longitudinal strain in patients receiving cardiotoxic chemotherapy; they derived accurate patient trajectories from this, providing improved prognostication as compared to conventional methods.

### 4.2 AI operated Echocardiography

Although AI has made significant forays into training, acquisition, interpretation and diagnostics of echocardiography, there remains the issue of operating the machine and probe. The solution likely lies in robotics. Arbeille *et al*. [60] first demonstrated the efficacy of robotics with a tele-operated motorized echocardiography probe controlled by trained sonographers on a cohort of 41 patients. They were able to generate similar measurements in 93–100% of cases, with no statistically significant difference (*p *> 0.05) [60]. Further to this, Boman *et al*. [61], randomized patients in a rural community in Sweden to a local robot-assisted remote echocardiogram vs. an echocardiogram at the nearest specialty hospital. They assessed process time and time from randomisation to specialist consultation and found these metrics were significantly reduced with the robotic option, they also found patient satisfaction to be improved in the robotic arm [61]. This provides the perfect avenue for full automation of the echocardiographic examination, and ML is well suited for the development of a suitable software.

## 5. Challenges

Despite the massive progress of AI in echocardiography, there remains various challenges in its widespread clinical implementation. Firstly, the inner workings of DL are poorly understood, it is often likened to a black box, which creates a degree of hesitancy in its uptake by clinicians [62, 63]. One could argue this is not entirely necessary if patient outcomes are improved, software and machines are used daily despite little to-no understanding of the complex mechanisms involved. Secondly all ML suffers from overfitting if the training data is limited, this is where the model is too specifically trained that it performs poorly in predictive use in the field [63, 64]. The solution to this problem is in providing larger training data sets, however this can be challenging and time consuming, requiring labelling of imaging, appropriate training and adequate expertise [10, 63].

Another limitation is the requirement for high quality training data, AI requires extensive data banks of high quality images to train the algorithm [10, 64]. When applied to sub-optimal real world imaging, this can often lead to impaired analysis, with some studies citing error ranges of 3–16% for view identification and quality control [65]. The solution for this requires ongoing training of algorithms with real world data to ensure efficacy with sub-optimal imaging.

The most important limitation of AI is the paucity of studies with robust clinical outcomes. Studies continue to compare AI derived measurements and findings to conventional techniques; to the best of our knowledge there are no clinical trials assessing the outcomes of AI application to patient care. It has become clear that AI can match (and in some cases out-perform) conventional techniques in echocardiography interpretation and analysis, thus the next step will be in assessing the outcomes of its application on patients in real world clinical settings. Evidence of improved outcomes would be required prior to integration into routine clinical practice.

Finally, there are significant legal and ethical liabilities associated with AI in echocardiography, as such any integration of AI will require extensive validation prior to regulatory approval [63, 66]. Moreover, significant sonographer and cardiologist supervision will likely be required. Resistance to AI due to fear of losing jobs should be put to rest, as it is clear the role of AI is to assist clinicians by improving accuracy, training, speed and workflow [10, 26, 65]. Undoubtedly, a collaborative multidisciplinary effort with engineers, computer scientists, sonographers and cardiologists will be necessary for the successful implementation of AI in echocardiography [63].

## 6. Conclusions

AI has the potential to transform echocardiography. Studies have illustrated its efficacy in training sonographers, improving image acquisition and analysing and interpreting scans. In this review we have highlighted that ML algorithms are able to assess LV function, RV function, quantify chamber and valvular measurements and improve diagnostics. The main benefit of AI is the unmatched efficiency and reproducibility it offers, a valuable tool to meet the global rise in demand for echocardiography. The role of DL is very encouraging, as it has the capacity to improve prognostication and develop new classification models for existing pathologies, a truly transformative feature.

There remain substantial barriers to the widespread application and implementation of AI in the clinical workspace. The most significant of these is the lack of data on real clinical outcomes, the practical litmus test for all novel clinical tools. The issues of ethics, poor understanding of AI, and dataset limitations are also notable. Nevertheless, the future of AI in echocardiography shows great promise, its clinical application is within reach, and it is poised to revolutionise modern echocardiography.

## Abbreviations

AI, Artificial intelligence; CCTA, Coronary computed tomographic angiography; CMR, Cardiac magnetic resonance; CNN, Convolutional neural network; DL, Deep learning; LV, Left ventricle; LVAD, Left ventricular assist device; LVEF, Left ventricular ejection fraction; ML, Machine learning; RV, Right ventricle; 2D, Two-dimensional; 3D, Three-dimensional.
